# Ovariotomy

**Published:** 1897-03

**Authors:** Thomas V. Simpson

**Affiliations:** N. W. T.


### OVARIOTOMY.

By Thomas V. Simpson, V.S.,
N. W. T.

On reading up the subject in most works that deal with the operation of ovariotomy I get a very limited amount of information from them, as they do not deal with the operation in a manner that would be of much use to an operator who had large numbers of cows on a single ranch or farm to spay.

The reason I introduced spaying into this district was because nearly every stockman had a number of aged cows that he wanted to get rid of, and, as most of the farmers or ranchers allow their bulls to run with the herd, the old cows continue to drop a calf every year until they get so old that they will never make beef. Another reason is because most of the calves are females, and the deficiency of steers could be overcome by spaying the heifers. Also unspayed cows running with a bunch of steers are the source of a great deal of annoyance during the period of heat.

Most of those I did were operated upon through the side, as I find it the quickest and most convenient method; moreover, it is quite as safe in the hands of a careful operator. All of those operated on by the flank method did exceedingly well, notwithstanding thirteen of the one-year-olds spayed during hot weather got badly flyblown, making it necessary to put them in a stable and thoroughly cleanse every wound of maggots and dead tissue. I took out all the dead tissue with a pair of forceps, and left a healthy, open, granulating wound, afterward dressing with a solution of a preparation of creolin. They had been flyblown nearly a week before it was noticed that the flies had been at the wounds. Also the seven aged cows spayed during hot weather got flyblown, and were treated in the same manner with satisfactory results. Of the thirty-eight operated on, two of the aged cows showed signs of heat a month or two after the wound was healed, but none of the others have shown any signs whatever.

The way I operate through the flank is as follows: I first cast the animal by putting a rope around the horns, or in the case of a mulley around the neck, and tying to a post; then take another rope somewhat longer (we use a lasso here), and pass it around the animal's body, and tie so as to form a slip-knot. When the knot is on, slide the rope back over the hindquarters and down to the hocks, and then draw the rope tight around the hocks. This proves to be an easy method of getting the two hindfeet into a rope. Then draw tight and the animal will fall. Then tie to a post opposite to the one the head is tied to. This stretches the animal out straight and puts her in a favorable position for operating. She must be laid on the right side, as the incision is made over the rumen on the left side. I generally tie another rope to the lower forefoot after the animal is down, and tie it to something so that she cannot get her forefeet under her and roll over.

Now for the operation. Two buckets are necessary before commencing to operate; one containing a solution of creolin, or some other good antiseptic, into which the instruments are placed, the other containing pure water and castile or some antiseptic soap.

The latter solution I use in case my hands get a little dirty between operations. The instruments necessary are a scalpel and a spaying écraseur or shears; also a good heavy needle and silk or linen thread. I use the linen, and find it quite good enough for cattle. The incision is commenced in the upper part of the flank on the left side about four inches downward and forward from the external angle of the ileum and continued for four inches downward. Make the incision through the skin and subcutaneous connective tissue first, then through the obliquus abdominis externus muscle until the fibres of the obliquus abdominis internus are seen running anglewise across. Now the scalpel is needed no more, as it is better to part the fibres of the internal oblique muscle with the fingers than to cut through. Gradually part the fibres until the peritoneum is reached; then by using the index-finger you can puncture the peritoneum without much trouble. Gradually enlarge the opening until the hand can pass in.

It should not be necessary to state that the hands must be perfectly clean before commencing to operate. Cut the finger-nails short, and thoroughly scrub the hands in a 1 to 1000 bichloride solution; then you need not be afraid of any abscesses or foul running wounds forming in the region where the operation was performed.

Finding the ovaries is the most difficult part of the whole operation. The right hand I find best to introduce. In very young animals the ovaries can be found in the anterior part of the pelvic cavity by first finding the uterus, which is no easy matter, and following it forward until the ovary is found at the end of the cornu. In old cows it is more easily found. The uterus is felt as a firm organ and can be easily followed up until the ovaries are found about four or five inches from the end of the cornu. It is preferable to remove the furthest ovary first.

After removal of the ovaries all that remains to be done is to cleanse the wound and put in two or three good strong sutures in the skin and external oblique muscle. The fibres of the internal oblique will come right back to place and unite in a very short time.

The after-treatment consists in allowing plenty of freedom and seeing that no flies irritate the wound. Pine-tar makes a good application after the wound is sutured, and will help to keep the flies away.

I forgot to state that before making the incision in the flank the hair should be shaved off either with the scalpel or a razor.

As to the time of the year to operate I prefer late in the spring or early in the summer, before the blue-flies make their appearance. One year old is about the proper age to operate on young cattle.

Annexed is a list of cows I operated on and a few notes added thereto.

| No. | Age. | Condition of animal. | Time of year. | Weather. | Method of operation. | Remarks. |
|---|---|---|---|---|---|---|
| 1 | 4 years | Fair | May 7 | Good | Side | Placed in stocks and operated on standing; did well. |
| 2 | 1 year | Good | June 5 | Moderate | Side | Did well. |
| 3 | 1 year | Good | June 5 | Moderate | Side | Did well. |
| 4 | 4 years | Good | June 5 | Moderate | Side | Showed signs of heat day before; did as well as the rest. |
| 5 to 13 | Aged | Poor | June 9 | Very wet | Side | Did extremely well. Two of these cows showed signs of heat about two months afterward. |
| 14 | 3 years | Good | June 25 | Good | Per vagina | Not much inclination to move freely for two or three days; afterward did well. |
| 15 | 3 years | Good | | | | |
| 16 | 6 years | Fair | | | | |
| 17 | 7 years | Fair | | | | |
| 18 | 2 years | Good | June 27 | Wet | Per vagina | Virgin; very hard to operate on; did well. |
| 19 to 31 | 1 year | Good | July 4 | Hot | Side | Got flyblown; did well; very easy to operate on. |
| 32 to 38 | Aged | Very poor | July 7 | Very hot | Side | Got flyblown; did well afterward. |

Where corn is fed unground to horses care should be used not to let them have it in a mouldy condition. A few years ago, at Poughkeepsie, N. Y., eighteen horses in one stable were fed damp corn which was invaded by white mould. All the horses were attacked with violent symptoms and eight of them died. Many other similar cases are reported.—*Orange Judd Farm.*

The *Therapeutic Gazette* recommends a drachm and a half of picric acid and three ounces of alcohol, to which two pints of distilled water are added, as a suitable and effective dressing for burns. Apply to the burned parts with absorbent-cotton, well soaked. Prick the blisters and apply the bandages lightly where possible. It eases pain and limits the tendency to suppuration by coagulating the albuminous exudation, the healing taking place under a scab consisting of epithelial cells hardened by picric acid.

Glycerin injected directly into the uterus hastens the expulsion of the contents, and is worthy of a trial in canine practice, especially among the bull-terrier class, where difficult parturition is ofttimes met with.

